# *Fhf2* gene deletion causes temperature-sensitive cardiac conduction failure

**DOI:** 10.1038/ncomms12966

**Published:** 2016-10-04

**Authors:** David S. Park, Akshay Shekhar, Christopher Marra, Xianming Lin, Carolina Vasquez, Sergio Solinas, Kevin Kelley, Gregory Morley, Mitchell Goldfarb, Glenn I. Fishman

**Affiliations:** 1 The Leon H Charney Division of Cardiology, New York University School of Medicine, 522 First Avenue, Smilow 801, New York, New York 10016, USA; 2 Hunter College of City University, Department of Biological Sciences, Room HN810, 695 Park Avenue, New York, New York 10065, USA; 3 Graduate Center of City University, Neuroscience Subprogram, 365 Fifth Avenue, New York, New York 10016, USA; 4 University of Sassari, Department of Biomedical Science, Viale San Pietro 43/c, 07100 Sassari, Italy; 5 Mouse Genetics Core, Icahn Medical Institute, 1425 Madison Avenue, New York, New York 10029, USA

## Abstract

Fever is a highly conserved systemic response to infection dating back over 600 million years. Although conferring a survival benefit, fever can negatively impact the function of excitable tissues, such as the heart, producing cardiac arrhythmias. Here we show that mice lacking fibroblast growth factor homologous factor 2 (FHF2) have normal cardiac rhythm at baseline, but increasing core body temperature by as little as 3 °C causes coved-type ST elevations and progressive conduction failure that is fully reversible upon return to normothermia. FHF2-deficient cardiomyocytes generate action potentials upon current injection at 25 °C but are unexcitable at 40 °C. The absence of FHF2 accelerates the rate of closed-state and open-state sodium channel inactivation, which synergizes with temperature-dependent enhancement of inactivation rate to severely suppress cardiac sodium currents at elevated temperatures. Our experimental and computational results identify an essential role for FHF2 in dictating myocardial excitability and conduction that safeguards against temperature-sensitive conduction failure.

## Abstract (web summary)

Fever is a defence mechanism against infection, but it may also cause abnormal heart rhythm *via* unknown mechanism. Here the authors identify FHF2 protein as a key regulator of myocardial excitability that protects the heart against conduction failure in response to an increase in body temperature.

## Graphical abstract

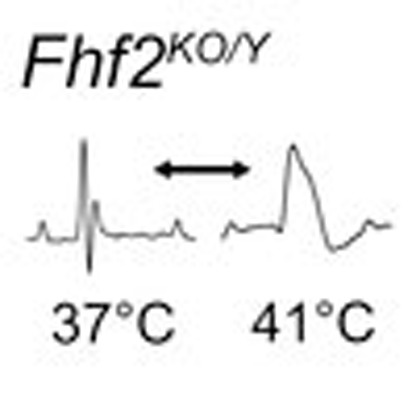

Fever-induced arrhythmias^1^ and seizures^2^ are well documented, and are often associated with mutations in sodium channels, suggesting that deficient sodium current reserve is an important determinant for electrical instability during hyperthermic states. Indeed, elevation in core body temperature by fever or external heating^3^ is a known trigger for ventricular fibrillation/malignant syncope in patients with Brugada syndrome (BrS)^4^, an inherited arrhythmia condition diagnosed by characteristic electrocardiographic (ECG) abnormalities in the right precordial leads. Loss of function mutations in *SCN5A*, which encodes the pore-forming subunit of the cardiac sodium channel Na_V_1.5, have been identified in ∼20% of BrS patients^5^. Biophysical analyses of mutant sodium channels from febrile BrS patients have not identified a unifying mechanism for the temperature-sensitive phenotype^6^,^7^. These data suggest that factors in addition to *SCN5A* are playing important roles in regulating the sodium current that ultimately predispose BrS patients to fever-induced arrhythmias.

FHFs, also termed iFGFs, are a family of proteins that bind to the cytoplasmic tails of voltage-gated sodium channels (VGSCs)^8^,^9^,^10^, modulating channel inactivation and cellular excitability^11^,^12^,^13^. We generated mice lacking fibroblast growth factor homologous factor 2 (*Fhf2*^*KO*^) to study its role in regulating cardiac excitability under normal and pathological states. *Fhf2*^*KO*^ mice have normal cardiac rhythm at baseline, but exhibit temperature-sensitive electrocardiographic changes, including coved-type ST elevations and progressive conduction failure that is fully reversible upon return to normal body temperature. Optical mapping reveals severe conduction slowing in mutant hearts at 37 °C that is further exacerbated by temperature elevation. FHF2-deficient cardiomyocytes generate action potentials upon current injection at 25 °C but are unexcitable at 40 °C. Loss of FHF2 results in a hyperpolarizing shift of steady-state inactivation of the sodium current and accelerates the rate of closed-state and open-state sodium channel inactivation, which synergizes with temperature-dependent enhancement of inactivation rate to severely suppress cardiac sodium currents at elevated temperatures. Our experimental and computational results demonstrate that FHF2 is a key regulator of myocardial excitability, protecting the heart against conduction failure under hyperthermic conditions.

## Results

### Derivation and validation of *Fhf2* ^*KO*^ mice

**Figure 1:**
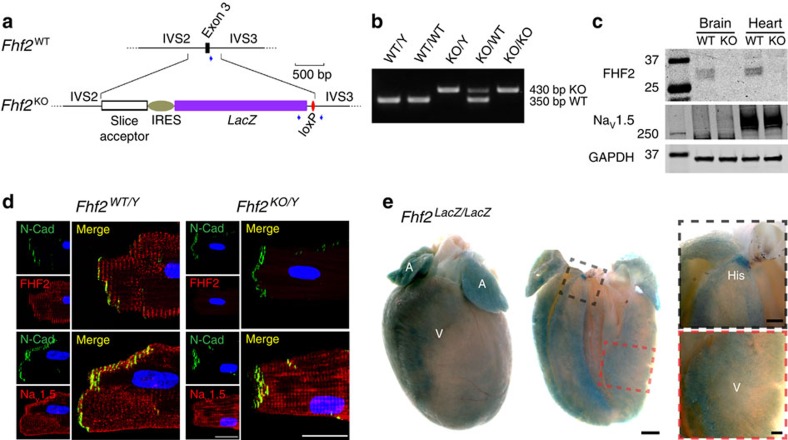
Derivation and validation of *Fhf2*^*KO*^ mice. (**a**) Schematic of *Fhf2*^*WT*^ and *Fhf2*^*KO*^ alleles. The *Fhf2*^*KO*^ allele differs from the *Fhf2*^*WT*^ allele by the replacement of a 570-base genomic segment spanning coding exon 3 with a cassette containing splice acceptor site, internal ribosome entry site, a β-galactosidase coding sequence and residual loxP site. Positions and orientations of three PCR primers are indicated (blue arrowheads). (**b**) PCR genotyping of *Fhf2*^*WT*^ and *Fhf2*^*KO*^ alleles. Simultaneous amplification of wild-type 350 bp and mutant 430 bp allelic segments were resolved on agarose gel. (**c**) Immunoblotting of *Fhf2*^*WT/Y*^ and *Fhf2*^*KO/Y*^ tissue extracts. 40 mcg protein from Triton X-100-soluble fractions of brain and heart lysates were electrophoresed and immunoblotted to detect FHF2 (top), Na_V_1.5 (middle) and GAPDH (bottom). The prominent ∼30 kDa species in *Fhf2*^*WT/Y*^ heart corresponding to the FHF2VY isoform are not detected in *Fhf2*^*KO/Y*^ samples. (**d**) Immunofluorescence detection of FHF2 and Na_V_1.5 in *Fhf2*^*WT/Y*^ and *Fhf2*^*KO/Y*^ cardiomyocytes. Dissociated cells were fixed and probed with antibodies to FHF2 (red), Na_V_1.5 (red) and N-cadherin (green) along with DAPI nuclear stain (blue). FHF2 and Na_V_1.5 colocalize to *Fhf2*^*WT/Y*^ striated T-tubules. Na_V_1.5 is comparably localized in the *Fhf2*^*KO/Y*^ myocyte, while FHF2 is not detected. (**e**) Whole-mount X-Gal staining of a *Fhf2*^*LacZ/LacZ*^ heart. LacZ at native sites of *Fhf2* expression in targeted alleles (**a**) was detectable throughout the heart, including atria (A), ventricles (V) and bundle of His (His). Higher magnification views of His bundle (black dashed box) and the left ventricular free wall (red dashed box). Scale, 25 μm (**d**); 1 mm (**e**, left); 250 μm (**e**, right).

We engineered mice bearing a deletion within the X-linked *Fhf2* gene (Fig. 1a,b) in order to test for possible effects on cardiac rhythm. The absence of FHF2 protein in *Fhf2*^*KO*^ mice was confirmed in immunoblots of heart and brain tissue (Fig. 1c), and by ventricular myocyte immunofluorescence (Fig. 1d). Notably, there was no noticeable change in Na_V_1.5 protein levels or subcellular localization in *Fhf2*^*KO*^ ventricular myocytes. The IRES-lacZ insertion within the *Fhf2*^*KO*^ allele (Fig. 1a) allowed for whole-mount X-Gal staining of the heart and demonstrated widespread *Fhf2* gene expression in atria, ventricles, and the His-Purkinje system (Fig. 1e).

### *Fhf2* ^*KO*^ mice exhibit hyperthermia-induced conduction defects

**Figure 2:**
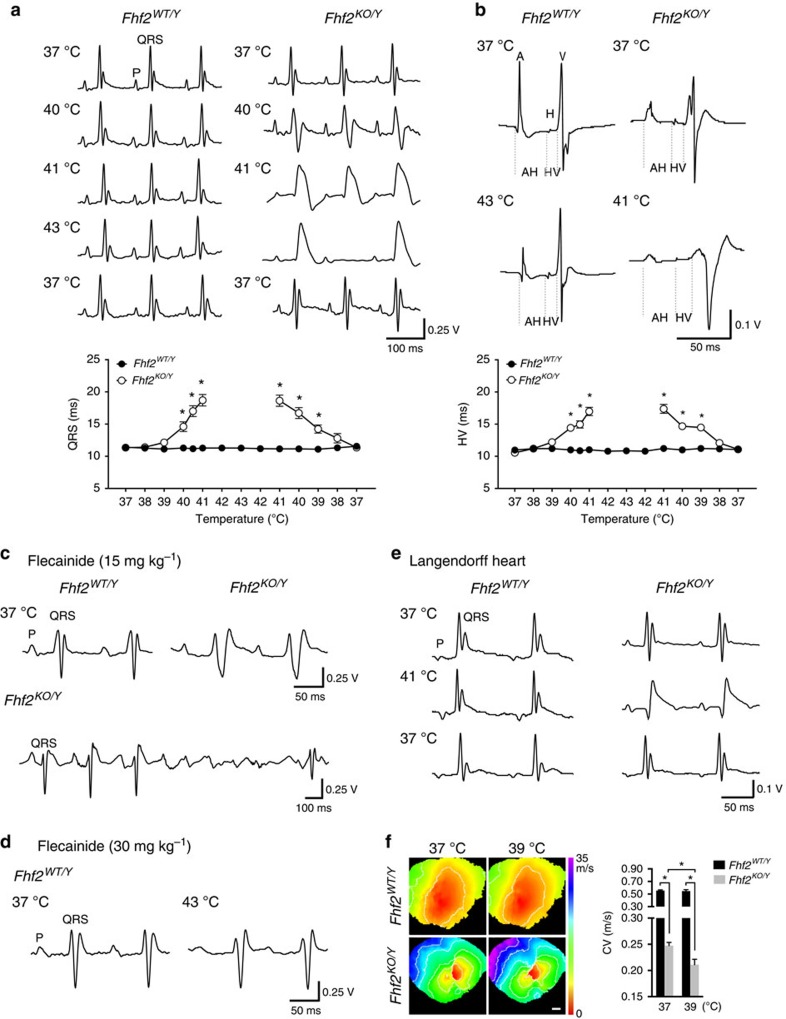
Hyperthermia-induced electrocardiographic (ECG) changes and conduction block in the *Fhf2*^*KO/Y*^ heart. (**a**) Representative surface ECG traces of adult (8–12 wk) *Fhf2*^*WT/Y*^ and *Fhf2*^*KO/Y*^ mice subjected to hyperthermia-induction protocol. External heat lamp was used to raise core body temperature from 37 to 43 °C followed by cooling to 37 °C (1 °C min^−1^). *Fhf2*^*KO/Y*^ ECG parameters were similar to *Fhf2*^*WT/Y*^ animals at 37 °C (*n*=10) but were significantly prolonged in mutant mice at elevated temperatures (≥40 °C) (*n*=9 mutants and 7 controls). Coved-type ST elevations and high degree AV block with periods of ventricular asystole were noted in *Fhf2*^*KO/Y*^ mice above 40 °C. *Fhf2*^*WT/Y*^ mice did not exhibit altered ECG parameters at any temperature (37–43 °C). QRS duration is plotted against core body temperature. (**b**) Representative intracardiac electrogram traces during hyperthermia-induction protocol. Intracardiac conduction intervals (AH, HV, AVI) of *Fhf2*^*KO/Y*^ were similar to *Fhf2*^*WT/Y*^ animals at 37 °C but were significantly prolonged in mutant mice (*n*=3) at elevated temperatures (≥40 °C). HV interval duration is plotted against core body temperature. (**c**) Flecainide (15 mg kg^−1^) challenge was performed in adult (8–12 wk) *Fhf2*^*WT/Y*^ and *Fhf2*^*KO/Y*^ mice (*n*=5). Representative surface ECG traces five minutes after administration of flecainide demonstrates significantly prolonged ECG parameters in *Fhf2*^*KO/Y*^ compared with *Fhf2*^*WT/Y*^ mice at 37 °C (upper panels). Representative surface ECG traces 15 min after administration of flecainide 15 mg kg^−1^ in *Fhf2*^*KO/Y*^ showing progressive heart block (lower panel). (**d**) Flecainide (30 mg kg^−1^) challenge was performed in adult (8–12 wk) *Fhf2*^*WT/Y*^ mice in combination with the hyperthermia-induction protocol. Representative surface ECG traces of flecainide treated *Fhf2*^*WT/Y*^ mice (*n*=5) at 37 °C and 43 °C. *Fhf2*^*WT/Y*^ mice treated with flecainide did not exhibit any further prolongation of conduction parameters with elevated temperature (37–43 °C). (**e**) Langendorff-perfused *Fhf2*^*WT/Y*^ and *Fhf2*^*KO/Y*^ whole hearts (*n*=4) subjected to hyperthermia-induction protocol. Volume-conducted ECG recordings of isolated *Fhf2*^*KO/Y*^ hearts recapitulated the *in vivo* findings. (**f**) Representative ventricular activation maps from *Fhf2*^*WT/Y*^ and *Fhf2*^*KO/Y*^ at 37 and 39 °C (*n*=3). Hearts were paced at a 200 ms basic cycle length. Isochrones are drawn 5 ms apart. AH, atrial-His; HV, His-ventricular; AVI, atrioventricular interval; CV, conduction velocity. Scale, 1 mm (**f**). Data represent mean±s.e.m. *significant *P* values, Student's *t*-test.

Female *Fhf2*^*KO/KO*^ and male *Fhf2*^*KO/Y*^ mice were viable and fertile. *Fhf2*^*KO/Y*^ mice exhibited normal baseline ECG parameters at 37 °C (Fig. 2a and Supplementary Table 1). Cardiac structural and functional assessments by transthoracic echocardiography were also normal under euthermic conditions (Supplementary Table 2). However, *Fhf2*^*KO/Y*^ mice were highly temperature-sensitive. Elevation of core body temperature by external heat source resulted in marked conduction slowing as evidenced by progressive P and QRS wave prolongation and atrioventricular (AV) block (Fig. 2a and Supplementary Table 1). Above 40 °C, all mutant mice developed coved-type ST elevations with T wave inversions, reminiscent of the Brugada pattern ECG (Fig. 2a). Mutant mice did not tolerate sustained temperature elevation due to high-grade AV block and progressive conduction failure. With subsequent cooling to 37 °C, all ECG conduction parameters returned to baseline. In contrast, male wild type (*Fhf2*^*WT/Y*^) mice displayed normal ECG parameters throughout the temperature ramp up to 43 °C (Fig. 2a and Supplementary Table 1). Measurement of intracardiac intervals including atrial-His (AH) and His-ventricular (HV) intervals, which are measures of AV nodal conduction time and His-Purkinje-mediated ventricular activation time, respectively, were similar to *Fhf2*^*WT/Y*^ mice at 37 °C but were significantly prolonged in mutant mice at elevated temperatures (Fig. 2b and Supplementary Table 1).

### *Fhf2* ^*KO*^ mice display increased sensitivity to flecainide

*Fhf2*^*KO/Y*^ animals were highly sensitive to the sodium channel blocker flecainide (15 mg kg^−1^), which caused marked P and QRS wave prolongation and AV block at 37 °C (Fig. 2c and Supplementary Table 3). In contrast, *Fhf2*^*WT/Y*^ mice were able to tolerate high dose flecainide (30 mg kg^−1^), and despite having marked prolongation of P, PR and QRS durations, the conduction parameters remained stable during temperature ramp up to 43 °C (Fig. 2d and Supplementary Fig. 1).

### Isolated *Fhf2* ^*KO*^ hearts retain temperature-sensitive defects

To rule out extra-cardiac contributions to the temperature-sensitive phenotype, including autonomic effects, explanted *Fhf2*^*KO/Y*^ and *Fhf2*^*WT/Y*^ hearts were Langendorff-perfused and subjected to a temperature ramp protocol with a maximum perfusion temperature of 43 °C (Fig. 2e). Similar to the *in vivo* findings, *Fhf2*^*KO/Y*^ hearts exhibited progressive P and QRS wave prolongation, and AV block on volume-conducted ECG with temperature elevation. Coved-type ST elevations with T wave inversions were again seen in mutant hearts at temperatures greater than 40 °C. *Fhf2*^*WT/Y*^ hearts displayed stable conduction parameters throughout the temperature recordings.

Optical mapping of *Fhf2*^*KO/Y*^ and *Fhf2*^*WT/Y*^ hearts using a voltage-sensitive dye was performed to evaluate differences in epicardial conduction velocities (CV) at 37 and 39 °C. Activation maps of the anterior wall were obtained at a pacing cycle length of 200 ms (Fig. 2f). Epicardial CV were markedly slower in *Fhf2*^*KO/Y*^ compared with *Fhf2*^*WT/Y*^ hearts at 37 °C (0.25±0.0063, m s^−1^ versus 0.56±0.01 m s^−1^, KO versus WT, respectively *P*=1.35E-5, Student's *t*-test). CV slowing was global and not confined to the right ventricular outflow tract, as has been reported in some patients with BrS (refs 14, 15, 16). *Fhf2*^*KO/Y*^ hearts showed further reduction in epicardial CV at 39 °C, while *Fhf2*^*WT/Y*^ hearts maintained stable epicardial CV measurements at higher temperature.

### *Fhf2* ^*KO*^ cardiomyocytes have reduced excitability at 40 °C

**Figure 3:**
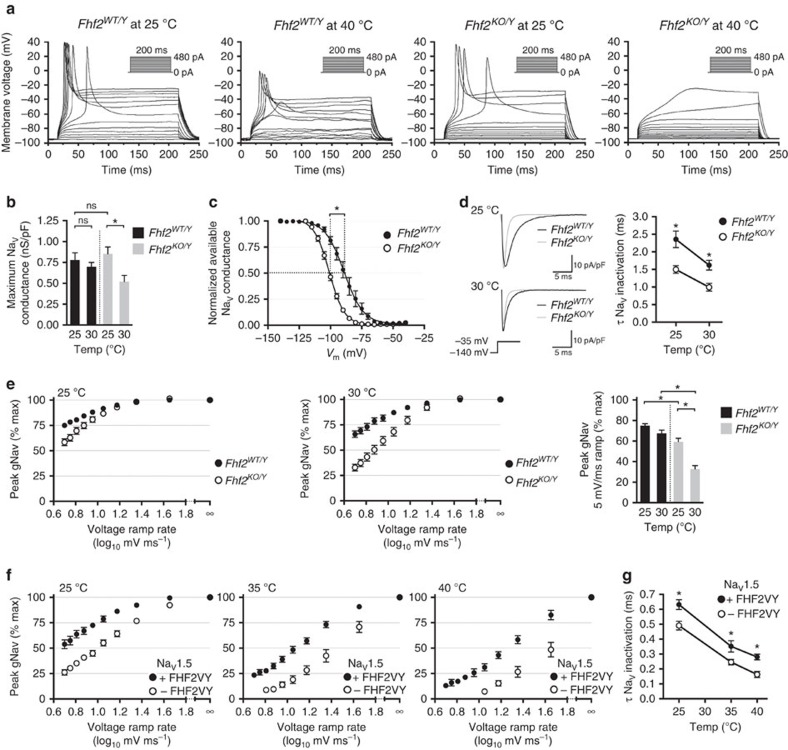
Temperature-dependent excitation failure and altered sodium channel gating in the absence of FHF2. (**a**) Ventricular cardiomyocyte excitability. Superimposed voltage traces of representative patched *Fhf2*^*WT/Y*^ and *Fhf2*^*KO/Y*^ cardiomyocytes injected with depolarizing current (0–480 pA in 40 pA steps) at 25 °C and in the same cells after raising temperature to 40 °C. All tested *Fhf2*^*KO/Y*^ cells were inexcitable at 40 °C. (**b**) Cardiomyocyte Na_V_ peak conductance (gNa_V_-peak). gNa_V_-peak following −120 mV to −35 mV depolarization step was measured for *Fhf2*^*WT/Y*^ and *Fhf2*^*KO/Y*^ cells at 25 °C and 30 °C. (**c**) Cardiomyocyte Na_V_ steady-state inactivation. Available gNa_V_ at 25 °C after 60 ms conditioning at −140 to −40 mV is expressed as fraction maximal gNa_V_. Vertical dashed lines indicate *V*_1/2_ inactivation in *Fhf2*^*WT/Y*^ and *Fhf2*^*KO/Y*^ cells. (**d**) Cardiomyocyte Na_V_ open-state inactivation rate. Superimposed representative traces (left) from *Fhf2*^*WT/Y*^ (black line) and *Fhf2*^*KO/Y*^ (grey line) cardiomyocytes at 25 °C (top) and 30 °C (bottom) following depolarization to −35 mV. Fast exponential time constant (*τ*) for decay of sodium current in response to voltage step to −35 mV are plotted for *Fhf2*^*WT/Y*^ and *Fhf2*^*KO/Y*^ cells at 25 and 30 °C (right). (**e**) Voltage ramp Na_V_ inactivation in cardiomyocytes. gNa_V_-peak in response to voltage ramps to −50 mV at different rates was expressed as percentage of gNa_V_-peak elicited by step depolarization (∞ ramp rate). Slower ramp rates decrease gNa_V_–peak through closed-state inactivation, which is sensitized by both elevated temperature and *Fhf2* mutation (histogram). See Supplementary Fig. 2 for representative conductance traces. (**f**) Voltage ramp Na_V_1.5 inactivation with and without FHF2VY. HEK cells transfected with Na_V_1.5±FHF2VY subjected to voltage ramps to −30 mV at different rates at 25, 35 and 40 °C. Na_V_1.5 closed-state inactivation is increased by slowed voltage ramp, temperature elevation and the absence of FHF2VY. See Supplementary Fig. 3 for representative conductance traces. (**g**) Na_V_1.5 open-state inactivation. Fast τ for Na_V_1.5 current decay was measured in cells±FHF2VY at 25, 35 and 40 °C. Elevated temperature and absence of FHF2VY each accelerate Na_V_1.5 open-state inactivation. Data represent mean±s.e.m. *significant *P* values (all *P* values in Supplementary Tables 4 and 5); ns, not significant; Student's *t*-test.

To study the effects of FHF2 on cellular excitability, we acutely dissociated ventricular myocytes prepared from *Fhf2*^*WT/Y*^ and *Fhf2*^*KO/Y*^ mice and tested their ability to generate action potentials (AP) at 25 and 40 °C in response to current injection through whole-cell patch. *Fhf2*^*WT/Y*^ cells could generate action potentials at either temperature, although AP waveforms were significantly attenuated at 40 °C (Fig. 3a and Supplementary Table 4). By contrast, *Fhf2*^*KO/Y*^ cells had reduced amplitude AP waveforms at 25 °C and failed to fire at 40 °C (Fig. 3a and Supplementary Table 4). We conclude that the *Fhf2*^*KO*^ temperature-dependent conduction defect reflects, at least in part, an underlying deficit in excitability of individual cardiomyocytes.

### Altered sodium channel inactivation in *Fhf2* ^*KO*^ cardiomyocytes

The basis of *Fhf2*^*KO*^ cellular excitation deficit was probed by recording sodium currents from voltage clamped *Fhf2*^*WT/Y*^ and *Fhf2*^*KO/Y*^ cardiomyocytes. Voltage step commands elicited similar transient sodium current densities in *Fhf2*^*WT/Y*^ and *Fhf2*^*KO/Y*^ cells at 25 °C, although peak currents in *Fhf2*^*KO/Y*^ cells fell by 37% when recorded at 30 °C (Fig. 3b and Supplementary Table 4). Loss of *Fhf2* altered sodium channel inactivation in several ways. Channels in *Fhf2*^*KO/Y*^ cells underwent steady-state inactivation at more hyperpolarized potential than did channels in *Fhf2*^*WT/Y*^ cells at 25 °C (Fig. 3c and Supplementary Table 4). *Fhf2* deficiency also accelerated the rate of sodium current decay when recorded at either 25 or 30 °C, with mutation and elevated temperature combining to produce the fastest rate of open-state inactivation (Fig. 3d and Supplementary Table 4). Sodium channel closed-state inactivation rates were assayed by comparing peak sodium conductance generated by voltage ramps to conductance generated by instantaneous voltage step. While longer ramp durations reduced peak sodium conductance under all conditions due to closed-stated channel inactivation, higher temperature and *Fhf2* mutation each enhanced the effect of ramp duration on channel availability, with *Fhf2* mutation and elevated temperature combining to yield the most severe reduction in availability (Fig. 3e, Supplementary Fig. 2 and Supplementary Table 4).

Sodium channel inactivation kinetics were further studied in human embryonic kidney (HEK) cells transfected to express cardiac sodium channel Na_v_1.5 with or without FHF2VY, the principal protein isoform of FHF2 expressed in mouse heart^13^. Patched HEK cells more easily survived temperature ramping than did cardiomyocytes allowing assessment of Na_v_1.5 inactivation parameters at 25, 35 and 40 °C often within the same cell. Steady-state voltage dependent Na_v_1.5 inactivation at 25 °C occurred with *V*_1/2_=−82.2±1.6 mV (*n*=7) in the presence of FHF2VY and *V*_1/2_=−93.5±0.9 mV (*n*=9) in the absence of FHF2 (*P<*0.0002, Student's *t*-test) (Supplementary Table 5). The absence of FHF2VY also increased the rate of Na_V_1.5 closed-state and open-state inactivation at all temperatures (Fig. 3f,g, Supplementary Fig. 3 and Supplementary Table 5). Most notably, even a 45 mV ms^−1^ voltage ramp at 40 °C caused a greater than 50% reduction in Na_v_1.5 peak conductance in the absence of FHF2, while more than 80% of channels remained available under these conditions in the presence of FHF2VY (Fig. 3f and Supplementary Fig. 3).

### Computational models of *Fhf2* ^*WT*^ and *Fhf2* ^*KO*^ cardiomyocytes

**Figure 4:**
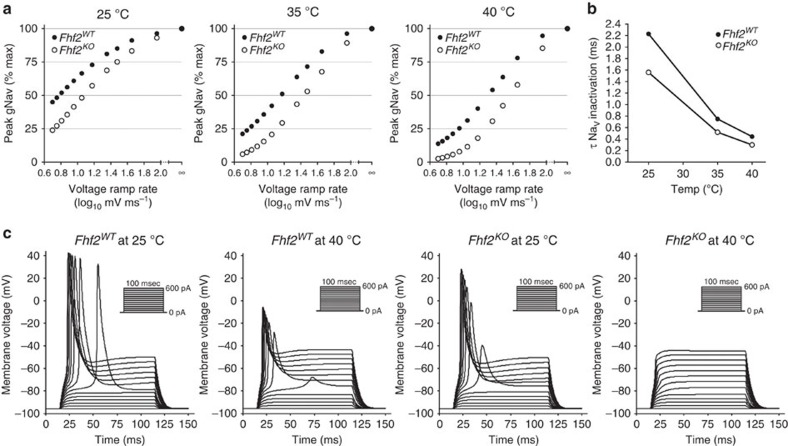
Computational models of *Fhf2*^*WT*^ and *Fhf2*^*KO*^ cardiomyocytes. The two models have the same gNa_V_–peak in response to instantaneous depolarization steps (Supplementary Fig. 4) and the same Na_V_ temperature-dependent kinetic scaling factor (Q10=3, see Methods), and differ only in their Na_V_ inactivation parameters. (**a**) Voltage ramp Na_V_ inactivation in cardiomyocyte models. Simulations were conducted at 25, 35 and 40 °C. Na_V_ sensitivity to closed-state inactivation during slower voltage ramp is potentiated in the *Fhf2*^*KO*^ model at all temperatures, closely resembling Na_V_1.5 recordings ±FHF2VY (Fig. 3f). (**b**) Na_V_ open-state inactivation rate. τ for decay of sodium current in response to voltage step to −20 mV are plotted for both models cells at 25, 35 and 40 °C. Open-state inactivation is accelerated in the *Fhf2*^*KO*^ model at all temperatures. (**c**) Model cardiomyocyte excitability. 100 ms current injections ranging from 0 to 600 pA (insets) were applied to *Fhf2*^*WT*^ and *Fhf2*^*KO*^ models at 25 and 40 °C. Both models are excitable at 25 °C, but only the *Fhf2*^*WT*^ model is excitable at 40 °C, comparable to cardiomyocyte recordings (Fig. 3a).

To establish whether hyperthermic excitation failure is attributable to altered cardiac sodium channel inactivation gating, we modified a previously generated mouse ventricular cardiomyocyte computational model^17^ to reflect the differential Na_v_ gating properties in the presence and absence of FHF2. The *Fhf2*^*WT*^ and *Fhf2*^*KO*^ myocyte models differed only in the open- and closed-state inactivation rate constants of the embedded Na_v_ 12-state Markov model. Both models generated the same peak Na_v_ conductance in response to step depolarization and the same voltage dependence of Na_v_ activation (Supplementary Fig. 4). But in comparison to the *Fhf2*^*WT*^ model, Na_v_ conductance in the *Fhf2*^*KO*^ model displayed a hyperpolarizing shift in the voltage dependence of steady state inactivation (Supplementary Fig. 4), exhibited faster closed-state inactivation in voltage ramp simulations (Fig. 4a), and faster open-state inactivation (Fig. 4b). Analogous to recorded channels, inactivation rates were temperature-dependent, such that *Fhf2* mutation and temperature elevation combined generated the fastest Na_v_ inactivation rates (Fig. 4a,b, Supplementary Fig. 4). Current injection simulations (Fig. 4c) demonstrated the consequences of altered Na_V_ inactivation gating. The *Fhf2*^*WT*^ model fired action potentials at both 25 and 40 °C, although AP waveforms were attenuated at higher temperature, while the *Fhf2*^*KO*^ model exhibited complete excitation failure at elevated temperature.

## Discussion

Preservation of cardiac sodium current density is critical for survival. Here we demonstrate that FHF2 acts to dampen the temperature-dependent acceleration of Na_V_1.5 open and closed-state inactivation and thereby maintain cardiac excitability and conduction throughout a range of physiologically relevant body temperatures. The importance of FHF2 becomes most apparent during hyperthermia when the intrinsic inactivation kinetics of Na_V_1.5, if left unchecked, can be brisk enough to severely impair sodium current density, leading to excitation block and conduction failure.

Although the ECG parameters of *Fhf2*^*KO*^ mice were normal at 37 °C, the degree of conduction slowing measured by optical mapping was substantial. This difference may reflect that *in vivo* ECGs, unlike the optical maps, were measured during sinus rhythm, where near simultaneous multi-site activation of the ventricular myocardium by the His-Purkinje system can mask myocardial conduction slowing. Purkinje myocytes express additional FHF isoforms^18^, which may confer some degree of protection against conduction slowing within the specialized conduction system in *Fhf2*^*KO*^ mice. The full extent of conduction slowing observed in the isolated heart preparations may also reflect the increased dependence of sodium current density on the slope of initial membrane depolarization in *Fhf2*^*KO*^ myocytes. In the intact heart, action potentials are triggered following non-instantaneous depolarization mediated by gap junctional currents passing from upstream cells^19^. Therefore, sodium current density in *Fhf2*^*KO*^ hearts should be exquisitely sensitive to conditions that increase intercellular resistivity between cardiomyocytes, such as the increase in interstitial volume that may be seen with Langendorff perfusion^20^. Indeed, the observation that FHF2 may modulate the dependency of sodium current density on junctional conductance has important implications for arrhythmia mechanisms associated with conditions that produce intercellular uncoupling, such as acute ischaemia^21^ or pathologic gap junction remodelling^22^.

The dynamic nature of the ECG abnormalities in *FHF2*^*KO*^ hearts is highly reminiscent of Brugada syndrome, where ECG parameters change from normal to coved-type ST elevations in the presence of fever or sodium channel blocking drugs. While the electrophysiological basis of the Brugada pattern ECG is controversial, some have reported focal conduction slowing in the right ventricular outflow tract as a potential mechanism^14^,^15^,^16^. Although optical mapping of *Fhf2*^*KO*^ hearts demonstrated global conduction slowing, additional analysis may be revealing. Furthermore, exploring the interplay between FHF2-dependent alterations in sodium current physiology and other ionic currents that have been implicated in the Brugada pattern ECG will certainly be of significant interest.

It should also be noted that in contrast to our results, Puranam *et al*.^23^ recently reported embryonic lethality in their lineage of *Fhf2*^*KO*^ mice. While the basis for this discrepancy is unclear, one possibility is that this other reported mutant allele was maintained on a C57Bl/6 background, with background strain influencing phenotype. Whether this lineage of *Fhf2*^*KO*^ mice survives to adulthood in other background strains should be evaluated.

In summary, our results identify the critical role of FHF2 in maintaining adequate sodium current reserve in response to hyperthermic stress. These results have direct implications for fever-induced Brugada syndrome, where loss of function sodium channel mutations may conspire with FHF-dependent mechanisms, such as allelic expression levels, to produce temperature-sensitive effects. It will be of interest to extend our studies in *Fhf2* mutant mice to additional fever models, such as challenge with lipopolysaccharide^24^ or cytokines^25^,^26^. It is also worth noting that the co-expression of FHFs and Na_V_s in the heart and brain may point to a unifying theory for both febrile arrhythmias and seizures^23^.

## Methods

### *Fhf2* ^*KO*^ mouse derivation

All protocols conformed to the Association for the Assessment and Accreditation of Laboratory Animal Care and the NYU School of Medicine Animal Care and Use Committee. A murine embryonic stem cell line bearing a ‘knockout first with conditional potential' cassette integrated into the *Fhf2* locus located on chromosome Xq26 (International Mouse Phenotype Consortium, clone EPD0339_4_F09) was injected into blastocysts to derive chimeric mice, which were outbred to establish viable mice bearing the targeted allele. Pronuclear injection of Cre recombinase-expressing plasmid into fertilized eggs of this lineage yielded progeny bearing Cre-mediated excision of *Fhf2* coding exon 3, which specifies an integral portion of the FHF β-trefoil fold^10^. The Cre-excised allele specifies bicistronic transcripts encoding truncated FHF2 peptides (from exons 1 to 2) and *E. coli* LacZ (Fig. 1a). A 3-primer polymerase chain reaction reaction with Phyre DNA polymerase allows simultaneous detection of wild-type (WT) and knockout (KO) alleles (Fig. 1a,b). Mice carrying the KO allele were backcrossed three generations to the 129/svPas strain before use in all cardiac physiology experiments.

### Antibody reagents

Immunofluorescence antibodies [target, dilution, (species, company)]. Primary antibodies: FHF2 (ref. 27) 1:500 (Epitope GGKSMSHNEST, Rabbit), Na_V_1.5 1:50 (mouse, Alomone, ASC-005), Sarcomeric Actinin 1:100 (mouse, Sigma, a7811), N-cad 1:100 (mouse, BD Biosciences, 610921). Mounting medium with DAPI (Vectashield, H-1200).

### Western blot antibodies

Primary antibodies: FHF2 1:1,000 (rabbit, Sigma, HPA002809); Na_V_1.5 1:500 (rabbit, Alomone Labs. ASC-005); GAPDH 1:750 (mouse, Millipore: mab374). Secondary antibodies: Goat anti-Rabbit 1:15,000 (Li-Cor 926-32211); Goat anti-Mouse 1:15,000 (Li-Cor 926-32220).

### Immunoblotting

Brain and exsanguinated heart tissues were homogenized in cold detergent-free buffer, after which membranes were solubilized by adding 1% Triton X-100. Clarified lysates were run on 4–20% precast polyacrylamide gradient gels (Invitrogen) and transferred to nitrocellulose (Bio-Rad) overnight at 4 °C. Nitrocellulose membranes were incubated in blocking buffer consisting of PBS (Na_V_1.5) or TBS (FHF2) with Tween-20 (0.05%) and 5% nonfat dry milk. Membranes were then incubated with specific primary antibodies diluted in 5% nonfat dry milk in PBST/TBST (0.05%) overnight at 4 °C followed by wash steps and secondary antibodies (Li-Cor). Antigen complexes were visualized and quantified with the Odyssey Imaging System (Li-Cor). The uncropped western blot images shown in Fig. 1c are displayed in Supplementary Fig. 5.

### Cardiomyocyte enzymatic dissociation experiments

Cardiac cells were dissociated from adult hearts that were Langendorff perfused and enzymatically digested according to AfCS Procedure Protocols PP00000125. Mice were heparinized (500 U kg^−1^) and killed with 100% carbon dioxide. Hearts were surgically removed *via* thoracotomy and immersed in ice cold perfusion buffer (composition (mmol l^−1^): 113 sodium chloride (NaCl), 4.7 potassium chloride (KCl), 0.6 potassium phosphate monobasic (KH_2_PO_4_), 0.6 sodium phosphate dibasic (Na_2_HPO_4_), 1.2 magnesium sulphate heptahydrate (MgSO_4_.7H_2_O), 12 sodium bicarbonate (NaHCO_3_), 10 potassium bicarbonate (KHCO3), 10 HEPES buffer solution, 30 mM taurine, 5.5 mM glucose and 10 2,3-Butanedione monoxime (BDM)). The aorta was cannulated and Langendorff perfused with perfusion buffer at a constant flow rate of 3 ml min^−1^ for 3 min. The perfusate was then switched to myocyte dissociation buffer (composition: 1 × perfusion buffer, 10 mg Liberase TM and 12.5 μM calcium chloride (CaCl_2_) at a constant flow rate of 3 ml min^−1^ for 8 min. Perfusate temperature was maintained at 37 °C. Heart was removed, placed in a dish containing 2.5 ml myocyte dissociation buffer plus 2.5 ml stop buffer 1 (composition: 1 × perfusion buffer, 10% bovine calf serum, and 12.5 μM CaCl_2_), and tissue above the atrioventricular ring was removed. Ventricles were teased into several small pieces with fine forceps. Cellular dissociation was achieved by further, gentle mechanical agitation *via* 15 ml transfer pipets. Quality of myocyte dissociation was then assessed by counting myocyte yield and percentage of rod-shaped myocytes. Only preparations with yields greater than 1 million cells and >60% rod-shaped myocytes were used for further experimentation. Cardiomyocytes were sedimented by centrifugation at 100*g* for 1 min in 15 ml tubes. The supernatant was discarded after sedimentation and the pellet was re-suspended in 10 ml of stop buffer 2 (composition: 1 × perfusion buffer, 5% bovine calf serum, and 12.5 μM calcium chloride (CaCl_2_)) added to increasing concentration of Ca^2+^ in 5 min intervals (+50 μl 10 mM Ca^2+^ (62 μM), +50 μl 10 mM Ca^2+^ (112 μM), +100 μl 10 mM Ca^2+^ (212 μM), +30 μl 100 mM Ca^2+^ (500 μM) and +50 μl 100 mM Ca^2+^ (1 mM)). Viability of myocytes was reassessed using the above quality control guidelines. Only cell preparations that passed these criteria were used for experimentation.

### Immunocytochemistry

Dissociated ventricular myocytes were fixed in 4%PFA for 5 min. Cells in suspension were spun onto slides using Shandon Cytospin II (Speed 250 r.p.m., 2 min). Cells were blocked with 10% serum and 0.01% Triton in PBS for 1 h, and then incubated with primary antibodies overnight. Cells were then washed in PBS and incubated with secondary antibodies conjugated to Alexa Fluor dyes (Invitrogen) for 1 h before mounting. Slides were coverslipped with Vectashield mounting media with DAPI (Vector Laboratories). Stained cells were visualized using a Leica TCS SP5 confocal microscope with Leica LAS AF acquisition software.

### Whole-mount staining for β-galactosidase activity

Tissues were collected in ice-cold PBS and perfusion fixed for 1 h in fix solution (2% formaldehyde, 0.2% glutaraldehyde, 0.02% Nonidet P-40 (NP-40), 0.01% sodium deoxycholate in PBS). After fixation, tissues were rinsed in PBS three times and then stained overnight at 37 °C in the dark with stain solution (5 mM K_3_Fe(CN)_6_, 5 mM K_4_Fe(CN)_6_, 1 mg ml^−1^ 5-bromo-4-chloro-3-indolyl-β-D-galactopyranoside (X-gal), 2 mM MgCl_2_, 0.02% NP-40, 0.01% sodium deoxycholate in PBS). Bright field images of hearts were taken using the Zeiss Discovery V8 microscope equipped with a Zeiss AxioCam Colour camera interfaced with Zeiss Zen 2012 software.

### Mouse *in vivo* electrophysiology and flecainide drug challenge

ECGs were obtained using subcutaneous electrodes attached at the four limbs (MP100, BIOPAC Systems). 8–12 week-old, male mice were anaesthetised with inhaled (2% v/v) isoflurane. Heart rate and core body temperature (rectal temperature probe) were continuously recorded. Flecainide (15 mg kg^−1^ or 30 mg kg^−1^) was administered *via* intraperitoneal injection. ECG analysis was performed in an unbiased fashion where 100 beats at each temperature endpoint were analysed using LabChart 7 Pro version 7.3.1 (ADInstruments, Inc). Detection and analysis of P wave, PR interval and QRS wave intervals were set to Mouse ECG parameters.

Intracardiac recordings were obtained in 8–12 week-old, male mice under isoflurane anaesthesia (1.5% v/v) using an octapolar catheter (EPR-800, Millar Instruments) placed *via* the right internal jugular vein for bipolar recordings at the level of the His bundle. Intracardiac intervals were measured from the His bundle catheter when the atrial, His, and ventricular electrograms were stable over >25 beats. Measurement of the AH interval was taken from the His bundle recording from the onset of deflection from baseline of the local atrial electrogram to the onset of deflection from baseline of the His bundle electrogram. Measurement of the HV interval was taken from the onset of deflection from baseline of the His bundle electrogram to the onset of deflection from baseline of the earliest ventricular electrogram, whether on surface ECG or intracardiac recordings. Core body temperature was initially maintained at 37 °C and gradually elevated up to 43 °C at a rate of 1 °C min^−1^ using an external heat lamp.

### Transthoracic echocardiography

Echocardiography was performed using the Vevo 2,100 high-resolution ultrasound imaging system with a real-time 30 MHz linear array scanhead (MS400) at a frame rate of 235 fps, a focal length of 8 mm, and a 10 × 10-mm field of view (Visualsonics; Toronto, Canada). 8–12 week-old, male mice were anaesthetised with 2% isofluorane, and hair was removed from the chest using a depilatory cream (Nair; Church & Dwight Co, Inc; Princeton, NJ). Warmed ultrasound transmission gel was placed on the chest and used to obtain left ventricular endpoints of cardiac function. B-mode cardiac imaging was conducted on transverse (short axis) plane. The papillary muscles were used for the short axis imaging landmark. M-mode recordings of the left ventricle were also recorded at the short axis B-mode imaging plane to obtain left ventricular function and dimensions through the cardiac cycle. Heart rate was monitored and core body temperature was maintained at 37.5 °C using a heated platform and a hair dryer throughout the procedure. Data analysis was performed on VisualSonics Vevo 2,100 V1.5.0 software (Visualsonics; Toronto, Canada). The following parameters were measured using three cardiac cycles from short axis M-mode images: diastolic and systolic left ventricular internal diameter, anterior wall thickness, and posterior wall thickness. From these measurements, left ventricular ejection fraction, per cent fractional shortening, stroke volume, and cardiac output were calculated within the Vevo software.

### Heart isolation and Langendorff perfusion

Male mice of 8–12 week-old were heparinized (500 U kg^−1^) and killed with 100% carbon dioxide. Hearts were surgically removed *via* a thoracotomy. While fully immersed in oxygenated (95% O_2_, 5% CO_2_) Tyrode's (composition (mmol l^−1^): NaCl 114, NaHCO_3_ 25, dextrose 10, KCl 4.6, CaCl_2_ 1.5, Na_2_PO_4_ 1.2, MgCl_2_ 0.7), the aorta was cannulated and Langendorff perfused at a constant pressure of 70 mm Hg. Perfusate temperature was initially maintained at 37 °C and increased by increments of 1 °C to a maximum temperature of 41 °C (optical mapping) or 43 °C (volume-conducted ECG). Perfusate temperature was allowed to reach steady state between temperature ramps.

### Optical mapping

High-resolution optical mapping experiments were performed as follows: excised hearts from 8 to 12 week-old, male mice were initially perfused with Tyrode's solution to clear blood and stabilize the heart, followed by Tyrode's solution containing 10 μM blebbistatin. Hearts were allowed to recover for 20 min and then stained with the voltage-sensitive dye, Di-4-ANEPPS (Molecular Probes Inc., Eugene, OR, USA). Light from green LEDs (530 nm; ThorLabs) was used as an excitation source and the emitted light (620 nm long pass) was detected with 1 high-resolution CMOS camera (Mi-CAM Ultima-L;SciMedia) at 1,000 frames per s in bin mode (100 × 100 pixels) with 14-bit resolution. Images were processed using a custom software package.

### Action potential recordings from adult cardiomyocytes

Excitability of acutely dissociated ventricular cardiomyocytes from *FHF2*^*WT/Y*^ and *FHF2*^*KO/Y*^ mice was conducted using a MultiClamp700 Amplifier, Digidata 1,440 analogue/digital converter and Clampex10 software (Molecular Devices). Cells were placed in the recording chamber under a Nikon Eclipse microscope and perfused with carbogen-bubbled bath solution (115 mM NaCl, 26 mM NaHCO_3,_ 3 mM KCl, 1.2 mM KH_2_PO_4_, 3 mM glucose, 2 mM myoinositol, 2 mM Na pyruvate, 7 mM HEPES, 1.2 mM Mg_2_SO_4_, 2 mM CaCl_2_, 0.2 mM CdCl_2_ at pH 7.2) maintained at 25 °C using an in-line heater (Warner Instruments). Patch pipettes were pulled with a P97 Micropipette Puller (Sutter Instruments) to yield a resistance of 1–1.5 MΩ when filled with 120 mM K gluconate, 4 mM NaCl, 5 mM KOH-buffered HEPES, 5 mM KOH-buffered EDTA, 15 mM glucose, 1 mM MgSO_4_, 3 mM Mg-ATP, 0.1 mM Na-GTP at pH 7.2. Pipette approach to cell was visualized using a 60 × water immersion lens and infrared illumination with differential interference contrast. Following tight seal formation and break-in to achieve whole-cell access, voltage clamp depolarization steps were used to confirm presence of large inward sodium current and measure capacitive current transients before switching to current clamp mode. Steadily applied negative current was used to set the amplifier-measured voltage to −85 mV, which was equal to −95 mV membrane potential due to a 10 mV junctional potential between pipette and bath solutions. Excitability was assessed by applying 200 ms injection sweeps of continuous current ranging from 0 to 480 pA in 40 pA steps. Membrane voltage recordings were high-pass filtered at 10 kHz and digitally acquired at 50 kHz. After data acquisition, the bath temperature was ramped to 40 °C over a 3–5 min time span, and excitability was retested with the same protocol. Only cells for which input resistance deviated less than 20% during temperature ramp were included in analysis.

### Na_v_1.5 currents in HEK cells

293T human embryonic kidney cells previously described in^28^, and displaying little or no endogenous sodium channel expression^29^ were transiently cotransfected with a 2:1 mixture of Na_v_1.5-expressing plasmid^30^, and a pIRES2-ZsGreen bicistronic plasmid (Clontech) expressing ZsGreen and mouse FHF2VY proteins. The same pIRES2-ZsGreen plasmid without FHF2 coding sequence served as control. Cells were trypsinized 3 h post-transfection, seeded onto gelatinized coverslips, and were used for recording after 48 h. For sodium current recordings, coverslips were transferred to recording chamber containing carbogen-buffered bath solution (115 mM NaCl, 26 mM NaHCO_3_, 3 mM KCl, 10 mM glucose, 4 mM MgCl_2_, 2 mM CaCl_2_, 0.2 mM CdCl_2_, 3 mM myoinositol, 2 mM Na pyruvate, 7 mM NaOH-buffered HEPES pH 7.2) at 25 °C and green fluorescent cells were whole-cell patched with pipettes filled with 104 mM CsF, 50 mM tetraethylamine chloride, 10 mM HEPES pH 7.2, 5 mM glucose, 2 mM MgCl_2_, 10 mM EGTA, 2 mM ATP, 0.2 mM GTP and having 1–2 MΩ resistance. For all recording protocols, sodium current was isolated during data acquisition by P/N subtraction of leak and capacitive currents (*N*=−6). To ensure voltage clamping during sodium channel activation was adequate, cells were subjected to a 19-sweep series of voltage steps from a hold of −120 mV to between −80 mV and +10 mV in 5 mV increments. As criterion for adequate clamp, transient current peaks for all voltage commands were nested within the larger current trace of a following or preceding voltage step command.

### Steady-state channel inactivation protocol

To determine the voltage dependence of steady state channel inactivation, a 19-sweep protocol used −120 mV holding command, a 60 ms variable test voltage step (−120+5(n−1) mV), followed by a −25 mV reporting pulse.

### Voltage ramp protocol

As a measure of closed state channel inactivation rate, a 10-sweep protocol used −120 mV hold command followed by depolarization to −30 mV either instantaneously (voltage step) or as a ramp ranging in time from 2 ms (=45 mV ms^−1^) to 18 ms (=5 mV ms^−1^).

### Temperature elevation

After recording at 25 °C, temperature was ramped to 35 °C and later to 40 °C. At elevated temperatures, the cell was first tested as above to ensure maintenance of tight clamp, after which the voltage ramp protocol was conducted. For many cells, voltage ramp protocols could be successfully run at all three temperatures (examples in Supplementary Fig. 3).

### Sodium current recordings in adult cardiomyocytes

All I_Na_ recordings in isolated cardiomyocytes were conducted in whole-cell configuration at 25 or 30 °C. Recording pipettes were filled with a solution containing (in mM): NaCl 5, CsF 135, EGTA 10, MgATP 5, HEPES 15, pH 7.2 with CsOH. Cells were maintained in a solution containing (in mM): NaCl 5, CsCl 112.5, TEACl 20, CdCl_2_ 0.1, MgCl_2_ 1, CaCl_2_, 1, HEPES 20, Glucose 11, pH 7.4 with CsOH. To determine the peak current voltage relation, 200 ms voltage pulses were applied to *V*_m_ −90 mV to +30 mV in 5 mV voltage steps, from a holding potential of *V*_m_=−120 mV. Interval between voltage steps was 3 s. Steady state inactivation was determined by stepping *V*_m_ to conditioning voltages of between −130 mV and −20 mV for 60 ms, followed by a 30 ms test pulse to *V*_m_=−20 mV to elicit *I*_Na_. The steady state voltage-dependent inactivation curves were fitted to Boltzmann's functions. As a measure of closed state channel inactivation rate, a 10-sweep protocol used −140 mV hold command followed by depolarization to −50 mV either instantaneously (voltage step) or as a ramp ranging in time from 2 ms (=45 mV ms^−1^) to 18 ms (=5 mV ms^−1^). All recordings were obtained utilizing an Axon multiclamp 700B Amplifier coupled to a pClamp system (versions 10.2, Axon Instruments, Foster City, CA).

### Cardiomyocyte excitation parameters

Spike threshold was defined as the point of accelerated upward voltage deflection at the base of a spike, mathematically defined as the voltage when d^2^*V*/dt^2^>0 as determined using Clampfit software (Molecular Devices).


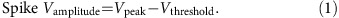


Values for *FHF2*^*WT/Y*^ versus *FHF2*^*KO/Y*^ were assessed for significant difference by two-tailed Student's *t* test. The number of *FHF2*^*WT/Y*^ versus *FHF2*^*KO/Y*^ cells that could spike and that failed to spike at 40 °C was used to assess significant difference by two-tailed Fisher Exact Probability test using an online calculator (http://vassarstats.net/odds2x2.html).

### Cardiomyocyte passive properties

Capacitance of each cell was measured as


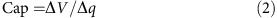


where Δ*q* is the current transient time integral to a negative voltage step command Δ*V*. Leak conductance was measured as


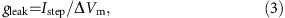


where Δ*V*_m_ is the steady-state change in membrane voltage in response to a negative current clamp command *I*_step_.

### Cardiomyocyte sodium conductance density

 


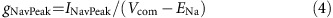


where *E*_Na_ is the sodium reversal potential and *I*_NavPeak_ is the peak inward current in response to a voltage command *V*_com_ within the linear ohmic range of the current/voltage relationship.

### Voltage dependence of steady state sodium channel inactivation

Peak sodium current accompanying the −25 mV reporting pulse was measured at each test voltage, and the fraction of channels available (not inactivated) at test voltage equalled *I*_Na-peak_(*V*_test_)/*I*_Na-peak_(maximum). To obtain *V*_1/2_ and *k* values for voltage dependence of steady state inactivation, current data were fitted to Boltzmann equation:


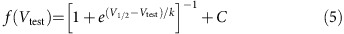


### Open-state sodium channel inactivation kinetics

Transient sodium current decay from 90% current peak to baseline was fitted to a two-term exponential decay function


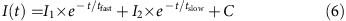


where *I*_1_ and *I*_2_ are the fast and slow decaying components of the transient current. The fast component *I*_1_ associated with decay constant *τ*_fast_ constituted between 70 and 100% of the total current in all analysed cells.

### Closed-state sodium channel inactivation kinetics

Data from voltage ramp protocols provided an estimate of closed-state channel inactivation rate as a function of voltage ramp rate. For each ramp command at each time point, we calculated


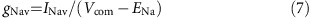


yielding sodium conductance traces as shown in Supplementary Fig. 3. Similarly,


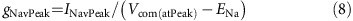


from which was calculated


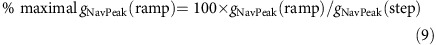


### Computational modelling

The *Fhf2*^*WT*^ and *Fhf2*^*KO*^ cardiomyocyte models were generated by introducing modifications to the murine ventricular cardiomyocyte model from Bondarenko *et al*.^17^. These changes included (1) adjusting membrane capacitance to 70 pF to more closely match our recorded measurements, (2) recalibrating the ‘leak' conductances by dividing the inward rectifying postassium conductance two-fold and replacing with an equal density of potassium conductance to achieve nonspiking responses to current injections similar to cardiomyocyte recordings (Fig. 4c), (3) changing the densities of ultrarapid-activating delayed rectifier and rapid-activating delayed rectifier potassium conductances to 0.112 mS μF^−1^ and 0.234 mS μF^−1^, respectively, in order to obtain more realistic post-spike repolarization and (4) replacing the fast sodium conductance with a 12-state Markov model sodium conductance:

This Na_V_ model is an adaptation of the 13-state model of Raman and Bean^31^, with the blocked open state and consequent resurgent current omitted. All rate constants were scaled by a thermodynamic factor


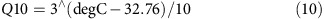


such that


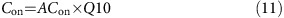



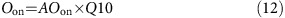



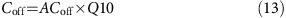



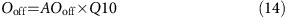



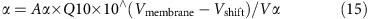



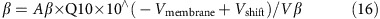



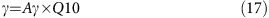



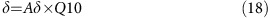


Parameter values shared by both *Fhf2*^*WT*^ and *Fhf2*^*KO*^ models are *Aα*=2.44375, ms^−1^, *Aβ*=0.01325, ms^−1^, *Aγ*=150 ms^−1^, *Aδ*=40 ms^−1^, *Vα*=*Vβ*=6 mV, *n*_1_=5.422, *n*_2_=3.279, *n*_3_=1.83, *n*_4_=0.738, *a*=(*O*_on_/*C*_on_)^0.25, *b*=(*O*_off_/*C*_off_)^0.25. For the *Fhf2*^*WT*^ model, *AC*_on_=0.001 ms^−1^, *AC*_off_=0.108 ms^−1^, *AO*_on_=1.05 ms^−1^, *AO*_off_=0.003 ms^−1^, and *V*_shift_=−58 mV, while in the *Fhf2*^*KO*^ model *AC*_on_=0.054 ms^−1^, *AC*_off_=0.027 ms^−1^, *AO*_on_=1.5 ms^−1^, *AO*_off_=0.001 ms^−1^, and *V*_shift_=−61 mV. These parameter differences preserved the voltage dependence of Na_V_ activation in the models while causing a 7 mV hyperpolarizing shift in voltage dependence of inactivation for the *Fhf2*^*KO*^ model (Supplementary Fig. 4) and accelerating closed-state and open-state inactivation rates (Fig. 4a,b) compared with the *Fhf2*^*WT*^ model.

Voltage–clamp and current–clamp simulations were run using the Myokit software interface^32^ and were initiated with a 200 ms channel state equilibration phase. Voltage–clamp simulations included single-step and double-step protocols to measure voltage dependence of Na_V_ activation and inactivation along with a ramp protocol comparable to that employed for experimental recordings to assess closed-state inactivation. For current-clamp simulations, an initial constant negative current was applied to adjust membrane voltage to −95 mV before adding positive current steps in multiples of 50 pA (0.7 pA pF^−1^).

### Statistics

Quantitative values are expressed as mean and standard error of the mean for each group. Two-tailed Student's *t* test was used to compare differences in continuous variables between mutant and control animals and cells. *P*<0.05 was considered statistically significant. Sample size calculations were done using preliminary data to design the experiment for measuring continuous variables. Groups were constructed to detect a 30% difference between experimental and control groups with a power of 90% and a significance level of 0.05. Experimental groups were blinded until the endpoints were analysed. Animal studies were done before genotyping ensuring blinded observations. The Prism statistical package (version 6, GraphPad) was used for analyses.

### Data availability

Cardiomyocyte computational model files are available for download at the ModelDB portal (https://senselab.med.yale.edu/modeldb/). The mouse lines will be made publicly available through a mouse repository. All other data and materials are available upon corresponding author request.

## Additional information

**How to cite this article:** Park, D. S. *et al*. *Fhf2* gene deletion causes temperature-sensitive cardiac conduction failure. *Nat. Commun.* **7,** 12966 doi: 10.1038/ncomms12966 (2016).

## Supplementary Material

Supplementary Information Supplementary Figures 1-5 and Supplementary Tables 1-5.
