# Supplementary material for: Fhf2 gene deletion causes temperature-sensitive cardiac conduction failure
Source: Nat Commun. 2016 Oct 4;7:12966. doi: 10.1038/ncomms12966 (PMC5059448; doi:10.1038/ncomms12966)
Supplement: Supplementary Information — Supplementary Figures 1-5 and Supplementary Tables 1-5. [file ncomms12966-s1.pdf]

SUPPLEMENTARY INFORMATION

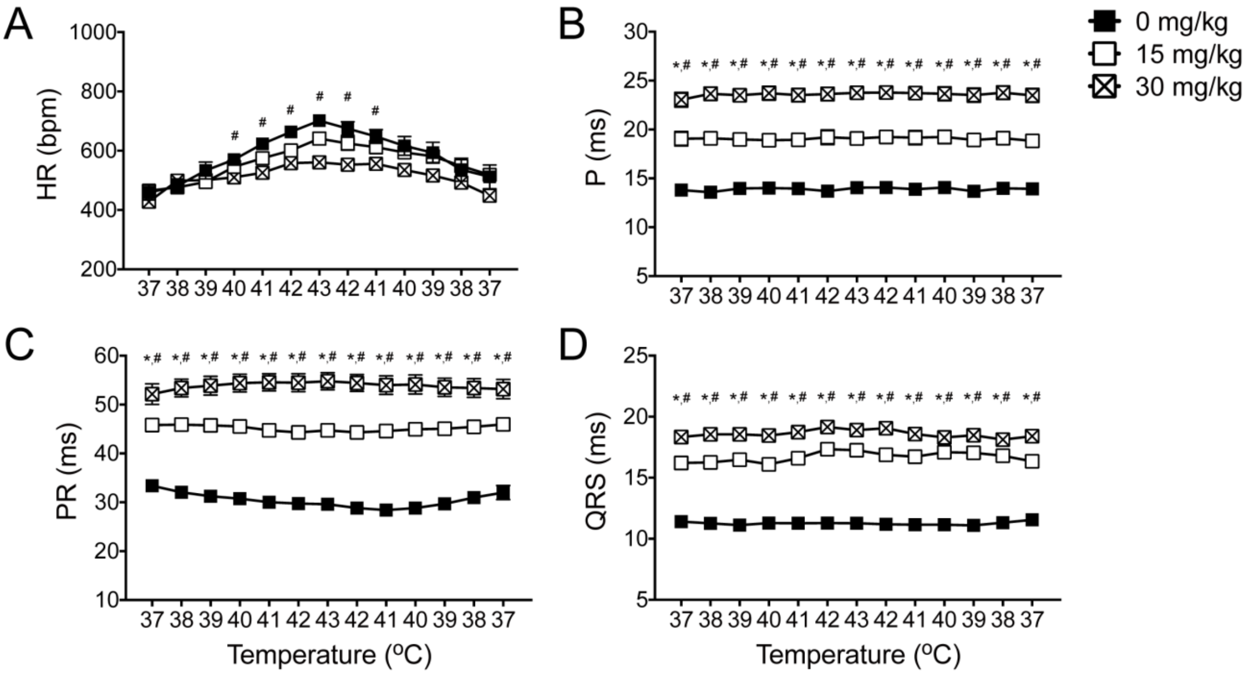


Supplementary Figure 1. Surface electrocardiograms (ECG) of flecainide challenged adult (8-12wk) *Fhf2*$^{WT/Y}$ mice during the hyperthermia-induction protocol. *Fhf2*$^{WT/Y}$ mice were administered flecainide (intraperitoneal) at 0, 15, or 30mg/kg ten minutes prior to hyperthermia-induction (n=5). Cardiac conduction intervals measured as HR, P wave duration, PR interval, and QRS duration are plotted with respect to core body temperature. ECG parameters remained stable during temperature ramp 37°C-43°C. HR; Heart rate. Data represents mean ± s.e.m. *$P<0.05$, 0mg/kg vs 15mg/kg; #P<0.05, 0mg/kg vs 30mg/kg, Student's t-test at each temperature.

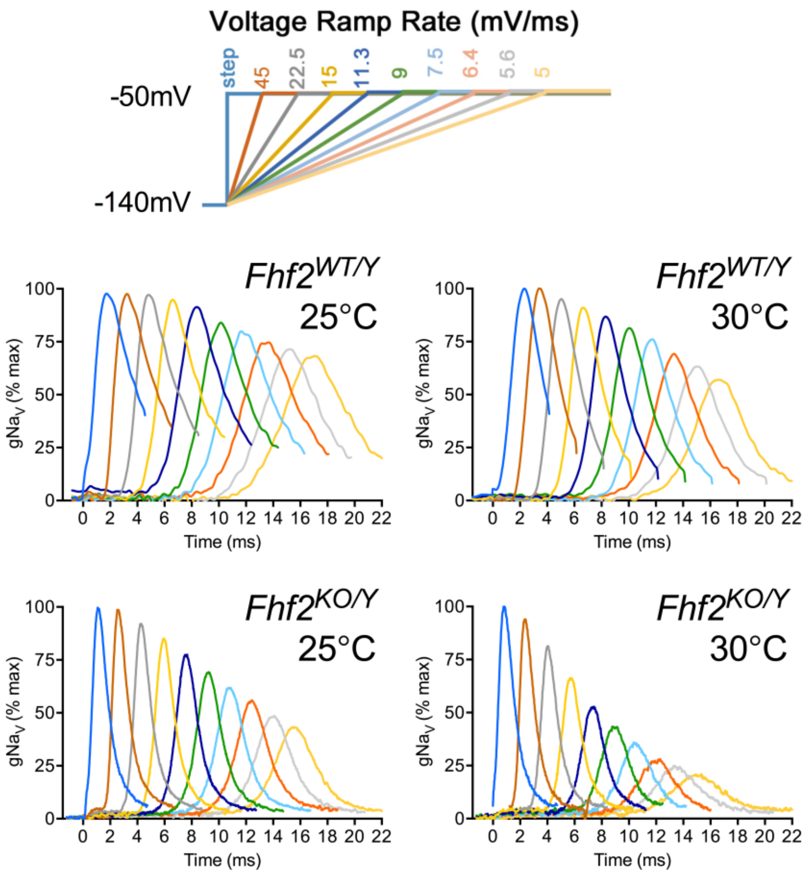


Supplementary Figure 2. Recorded cardiomyocyte $Na_V$ conductances during voltage ramp protocol. Cardiomyocytes were depolarized from -140 mV to -50 mV either instantaneously (voltage step) or at different ramp speeds while sodium currents were recorded. The voltage command for each ramp rate is shown color coded (top panel). Recorded current were converted to conductance offline and plotted as a percentage of maximum conductance. Middle panels correspond to a representative cardiomyocytes trace from *Fhf2*$^{WT/Y}$ heart recorded at 25°C and 30°C, while lower panels correspond to a representative cardiomyocytes trace from an *Fhf2*$^{KO/Y}$ heart.

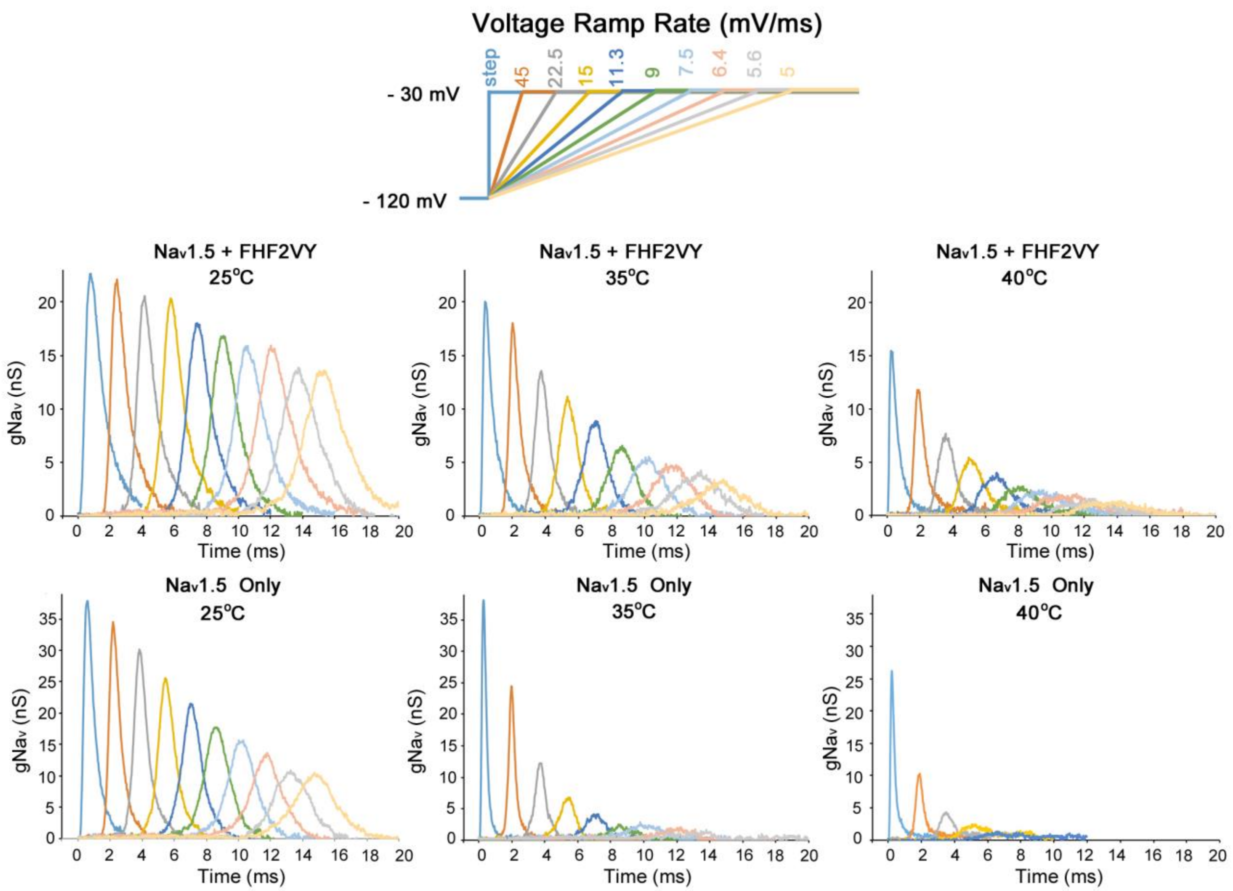


Supplementary Figure 3. Recorded HEK Na$_V$1.5 conductances during voltage ramp protocol. Cells were depolarized from -120 mV to -30 mV either instantaneously (voltage step) or at different ramp speeds while Na$_V$1.5 currents were recorded. The voltage command for each ramp rate is shown color coded (top panel). Recorded current were converted to conductance offline. Middle panels correspond to a single cell expressing Na$_V$1.5 + FHF2VY recorded at 25$^{o}$C, 35$^{o}$C, and 40$^{o}$C, while lower panels correspond to a single cell expressing Na$_V$1.5 without FHF2 recorded at all temperatures.

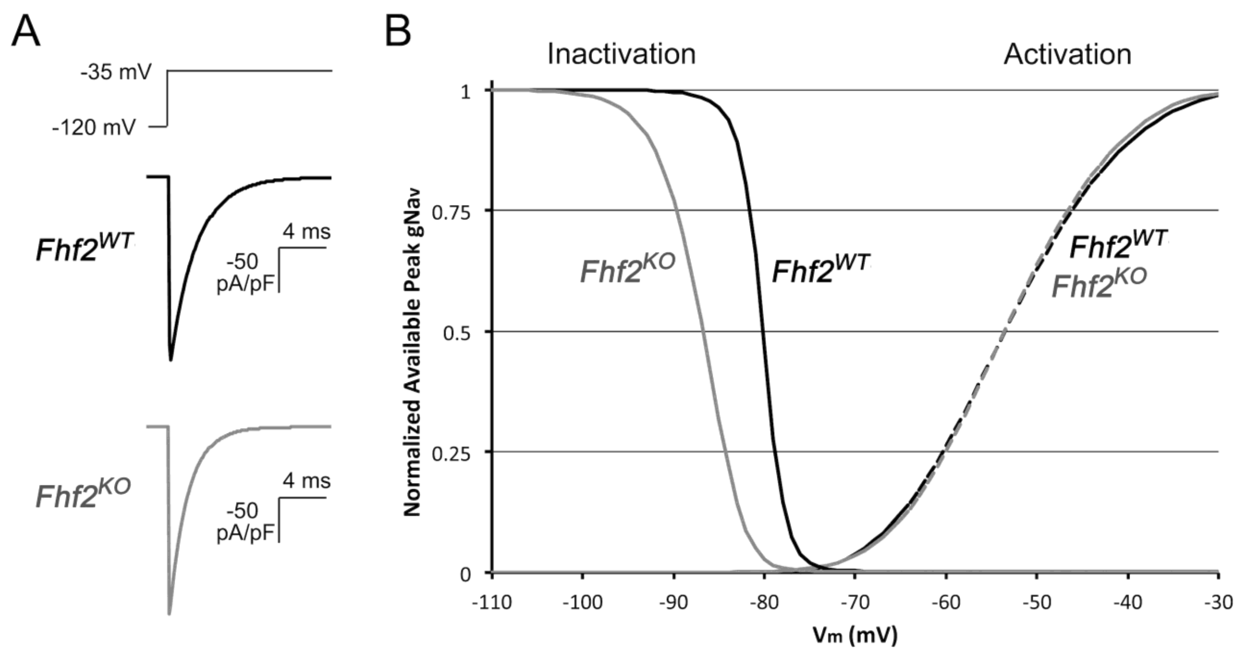


Supplementary Figure 4. Additional NaV properties in *Fhf2*$^{WT}$ and *Fhf2*$^{KO}$ cardiomyocyte models. A) Peak transient sodium current. NaV current traces in response to step depolarization (-120 mV to -35 mV) have the same peak amplitude in *Fhf2*$^{WT}$ and *Fhf2*$^{KO}$ models at 25°C, with current decaying faster in the *Fhf2*$^{KO}$ model. B) Voltage dependence of NaV activation and inactivation. NaV steady state inactivation in the *Fhf2*$^{KO}$ model occurs at more negative potential than in the *Fhf2*$^{WT}$ model, while activation of peak NaV conductance have same voltage dependence in both models.

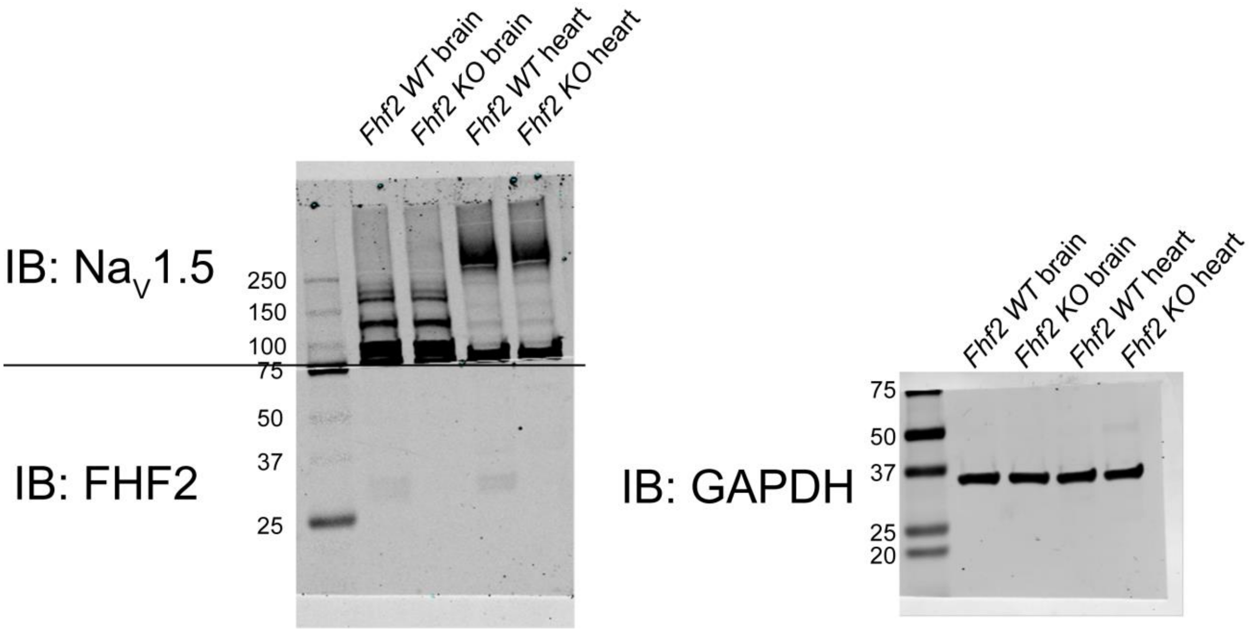


Supplementary Figure 5. Full western blots in Figure 1C.

Cardiac conduction intervals in *Fhf2*$^{WT/Y}$ and *Fhf2*$^{KO/Y}$ mice

| Temp (°C) | | ECG | | | | IEGM | | | |
|---|---|---|---|---|---|---|---|---|---|
| | | HR (bpm) | PR (ms) | P (ms) | QRS (ms) | HR (bpm) | AH (ms) | HV (ms) | AVI (ms) |
| 37 | *Fhf2*$^{WT/Y}$ (10,3) | 463 ± 17 | 33.9 ± 0.9 | 13.8 ± 0.2 | 11.2 ± 0.2 | 474 ± 4 | 22.9 ± 0.4 | 10.4 ± 0.3 | 33.2 ± 0.6 |
| | *Fhf2*$^{KO/Y}$ (10,3) | 469 ± 15 | 35.3 ± 0.7 | 13.9 ± 0.3 | 11.7 ± 0.3 | 480 ± 13 | 23.5 ± 0.5 | 10.3 ± 0.3 | 33.8 ± 0.4 |
| 38 | *Fhf2*$^{WT/Y}$ (7,3) | 481 ± 24 | 32.0 ± 1.2 | 13.6 ± 0.4 | 11.3 ± 0.4 | 499 ± 7 | 22.6 ± 0.3 | 10.9 ± 0.2 | 33.6 ± 0.3 |
| | *Fhf2*$^{KO/Y}$ (9,3) | 505 ± 14 | 33.7 ± 0.9 | 14.3 ± 0.3 | 11.4 ± 0.3 | 499 ± 8 | 22.7 ± 0.5 | 10.9 ± 0.2 | 33.7 ± 0.3 |
| 39 | *Fhf2*$^{WT/Y}$ (7,3) | 533 ± 28 | 31.3 ± 1.1 | 14.0 ± 0.5 | 11.1 ± 0.3 | 499 ± 8 | 21.4 ± 0.8 | 11.0 ± 0.2 | 32.4 ± 0.9 |
| | *Fhf2*$^{KO/Y}$ (9,3) | 537 ± 18 | 32.9 ± 0.9 | 14.1 ± 0.4 | 12.1 ± 0.4 | 509 ± 12 | 21.9 ± 0.6 | 11.9 ± 0.2* | 33.8 ± 0.4 |
| 40 | *Fhf2*$^{WT/Y}$ (7,3) | 570 ± 20 | 30.7 ± 0.9 | 14.0 ± 0.4 | 11.3 ± 0.3 | 518 ± 10 | 20.4 ± 1.0 | 10.6 ± 0.3 | 31.0 ± 0.8 |
| | *Fhf2*$^{KO/Y}$ (9,3) | 560 ± 16 | 34.4 ± 1.5 | 15.1 ± 0.6 | 14.6 ± 0.7** | 534 ± 12 | 23.1 ± 1.1 | 14.1 ± 0.2*** | 37.2 ± 1.3* |
| 40.5 | *Fhf2*$^{WT/Y}$ (7,3) | 591 ± 22 | 30.5 ± 1.2 | 13.6 ± 0.4 | 11.2 ± 0.3 | 546 ± 24 | 19.4 ± 0.5 | 10.7 ± 0.2 | 30.1 ± 0.7 |
| | *Fhf2*$^{KO/Y}$ (9,3) | 577 ± 18 | 34.1 ± 1.0* | 15.6 ± 0.7* | 17.0 ± 0.8*** | 550 ± 9 | 25.3 ± 0.7** | 14.5 ± 0.5** | 39.8 ± 0.4*** |
| 41 | *Fhf2*$^{WT/Y}$ (7,3) | 623 ± 20 | 30.0 ± 1.2 | 14.0 ± 0.6 | 11.3 ± 0.4 | 587 ± 15 | 19.3 ± 0.6 | 10.9 ± 0.2 | 30.2 ± 0.8 |
| | *Fhf2*$^{KO/Y}$ (9,3) | 595 ± 19 | 34.6 ± 1.3* | 16.5 ± 0.9* | 18.7 ± 0.9*** | 575 ± 15 | 25.4 ± 0.7** | 16.5 ± 0.5*** | 41.9 ± 1.0*** |
| 42 | *Fhf2*$^{WT/Y}$ (7,3) | 664 ± 19 | 29.7 ± 1.1 | 13.7 ± 0.6 | 11.3 ± 0.3 | 665 ± 28 | 19.1 ± 0.2 | 10.7 ± 0.3 | 29.8 ± 0.4 |
| | *Fhf2*$^{KO/Y}$ (9,3) | Lethal Temperature | | | | | | | |
| 43 | *Fhf2*$^{WT/Y}$ (7,3) | 701 ± 18 | 29.6 ± 0.9 | 14.0 ± 0.5 | 11.3 ± 0.4 | 680 ± 21 | 19.0 ± 0.4 | 10.6 ± 0.2 | 29.6 ± 0.6 |
| | *Fhf2*$^{KO/Y}$ (9,3) | Lethal Temperature | | | | | | | |
| 42 | *Fhf2*$^{WT/Y}$ (7,3) | 675 ± 23 | 28.8 ± 0.8 | 14.1 ± 0.5 | 11.2 ± 0.2 | 659 ± 13 | 19.9 ± 0.3 | 10.7 ± 0.3 | 30.5 ± 0.6 |
| | *Fhf2*$^{KO/Y}$ (9,3) | Lethal Temperature | | | | | | | |
| 41 | *Fhf2*$^{WT/Y}$ (7,3) | 648 ± 23 | 28.4 ± 0.7 | 13.9 ± 0.5 | 11.2 ± 0.2 | 652 ± 18 | 20.9 ± 0.6 | 11.0 ± 0.3 | 31.9 ± 0.5 |
| | *Fhf2*$^{KO/Y}$ (9,3) | 596 ± 19 | 34.5 ± 1.2** | 16.3 ± 0.9* | 18.6 ± 0.9*** | 587 ± 28 | 25.4 ± 0.4* | 16.8 ± 0.6*** | 42.3 ± 0.7* |
| 40 | *Fhf2*$^{WT/Y}$ (7,3) | 616 ± 32 | 28.8 ± 0.9 | 14.1 ± 0.4 | 11.2 ± 0.2 | 611 ± 16 | 20.9 ± 0.7 | 11.0 ± 0.4 | 31.8 ± 0.8 |
| | *Fhf2*$^{KO/Y}$ (9,3) | 543 ± 34 | 34.9 ± 1.0*** | 15.2 ± 0.7 | 16.7 ± 0.8*** | 543 ± 17 | 24.4 ± 0.4** | 14.3 ± 0.2** | 38.7 ± 0.3** |
| 39 | *Fhf2*$^{WT/Y}$ (7,3) | 593 ± 35 | 29.7 ± 0.7 | 13.7 ± 0.4 | 11.1 ± 0.3 | 595 ± 7 | 21.1 ± 0.6 | 10.9 ± 0.2 | 32.0 ± 0.7 |
| | *Fhf2*$^{KO/Y}$ (9,3) | 537 ± 18 | 34.5 ± 1.2** | 15.3 ± 0.7 | 14.2 ± 0.6** | 520 ± 12 | 23.1 ± 0.6 | 14.1 ± 0.2*** | 37.2 ± 0.8** |
| 38 | *Fhf2*$^{WT/Y}$ (7,3) | 536 ± 39 | 31.0 ± 1.1 | 14.0 ± 0.3 | 11.3 ± 0.3 | 552 ± 16 | 21.9 ± 0.6 | 10.9 ± 0.1 | 32.7 ± 0.6 |
| | *Fhf2*$^{KO/Y}$ (9,3) | 525 ± 15 | 34.1 ± 1.1 | 15.2 ± 0.7 | 12.8 ± 0.7 | 512 ± 6 | 21.8 ± 0.4 | 11.8 ± 0.1** | 33.6 ± 0.5 |
| 37 | *Fhf2*$^{WT/Y}$ (7,3) | 512 ± 40 | 32.0 ± 1.5 | 13.9 ± 0.3 | 11.6 ± 0.3 | 546 ± 17 | 22.2 ± 0.3 | 10.8 ± 0.1 | 33.0 ± 0.4 |
| | *Fhf2*$^{KO/Y}$ (9,3) | 485 ± 14 | 34.1 ± 0.9 | 14.0 ± 0.5 | 11.3 ± 0.4 | 493 ± 5 | 22.5 ± 0.8 | 10.8 ± 0.1 | 33.3 ± 0.8 |

Supplementary Table 1: Surface and intracardiac electrograms (IEGM) were obtained in adult *Fhf2*$^{WT/Y}$ and *Fhf2*$^{KO/Y}$ mice (8-12wks) at baseline (37°C) and during temperature ramp. Numbers in parentheses indicate the number of mice recorded for surface and IEGM values, respectively. Temp, Temperature; HR, Heart rate; AH, atrial-His interval; HV, His-ventricular interval; AVI, Atrioventricular interval. Data represents mean ± s.e.m. *P<0.05, **P<0.01, ***P<0.001, Student's t-test comparing *Fhf2*$^{KO/Y}$ vs *Fhf2*$^{WT/Y}$ at each temperature.

Echocardiographic parameters in *Fhf2*$^{WT/Y}$ and *Fhf2*$^{KO/Y}$ mice

| | HR (bpm) | EF (%) | FS (%) | SV (ul) | CO (ml/min) | LVAW,d (mm) | LVAW,s (mm) | LVPW,d (mm) | LVPW,s (mm) |
|---|---|---|---|---|---|---|---|---|---|
| *Fhf2*$^{WT/Y}$ (5) | 433 ± 21 | 61.9 ± 3.2 | 33.1 ± 2.1 | 48.2 ± 1.8 | 21.0 ± 1.6 | 0.82 ± 0.02 | 1.28 ± 0.02 | 0.82 ± 0.01 | 1.25 ± 0.02 |
| *Fhf2*$^{KO/Y}$ (6) | 445 ± 9 | 65.2 ± 1.4 | 35.4 ± 1.1 | 49.7 ± 5.0 | 22.1 ± 2.1 | 0.84 ± 0.02 | 1.25 ± 0.02 | 0.83 ± 0.01 | 1.22 ± 0.02 |

Supplementary Table 2: Left ventricular cardiac function and wall thickness were obtained in adult *Fhf2*$^{WT/Y}$ and *Fhf2*$^{KO/Y}$ mice (8-12wks) at baseline (37°C) via echocardiography. Numbers in parentheses indicate the number of mice recorded. HR (bpm), heart rate in beats per minute; EF, ejection fraction; FS, fractional shortening (short axis); SV, stroke volume; CO, cardiac output; LV, left ventricle; AW, anterior wall; PW, posterior wall; d, diastole; s, systole. Data represents mean ± s.e.m.

Flecainide challenge test: Cardiac conduction intervals in *Fhf2*$^{WT/Y}$ and *Fhf2*$^{KO/Y}$ mice

| | | ECG | | | |
|---|---|---|---|---|---|
| **Flecainide (mg/kg)** | | **HR (bpm)** | **PR (ms)** | **P (ms)** | **QRS (ms)** |
| 0 | *Fhf2*$^{WT/Y}$ (10) | 463 ± 17 | 33.9 ± 0.9 | 13.8 ± 0.2 | 11.2 ± 0.2 |
| | *Fhf2*$^{KO/Y}$ (10) | 469 ± 15 | 35.3 ± 0.7 | 13.9 ± 0.3 | 11.7 ± 0.3 |
| 15 | *Fhf2*$^{WT/Y}$ (5) | 433 ± 16 | 45.8 ± 1.2 | 19.1 ± 0.7 | 16.2 ± 0.4 |
| | *Fhf2*$^{KO/Y}$ (5) | 467 ± 20 | 53.9 ± 1.5** | 25.5 ± 1.2** | 24.6 ± 0.6*** |
| 30 | *Fhf2*$^{WT/Y}$ (5) | 464 ± 21 | 50.8 ± 0.9 | 22.6 ± 0.6 | 18.3 ± 0.5 |
| | *Fhf2*$^{KO/Y}$ (0) | Lethal Dose | | | |

Supplementary Table 3: Surface electrocardiograms were obtained in adult *Fhf2*$^{WT/Y}$ and *Fhf2*$^{KO/Y}$ mice (8-12wks) during flecainide challenge. Number in parentheses indicates the number of mice recorded. HR; Heart rate. Data represents mean ± s.e.m. ***P<0.001, Student's t-test comparing *Fhf2*$^{KO/Y}$ vs *Fhf2*$^{WT/Y}$ at each dosage.

| | 25$^o$C | | | | | 30$^o$C | | | | | 40$^o$C | | | | |
|---|---|---|---|---|---|---|---|---|---|---|---|---|---|---|---|
| | *$Fhf2^{WT}$* | | *$Fhf2^{KO}$* | | | *$Fhf2^{WT}$* | | *$Fhf2^{KO}$* | | | *$Fhf2^{WT}$* | | *$Fhf2^{KO}$* | | |
| **Parameter** | **mean +/- s.e.** | **# Cells** | **mean +/-s.e.** | **# Cells** | ***P (<)*** | **mean +/- s.e.** | **# Cells** | **mean +/-s.e.** | **# Cells** | ***P (<)*** | **mean +/- s.e.** | **# Cells** | **mean +/-s.e.** | **# Cells** | ***P (<)*** |
| AP Generation (% Cells) | 100 | (9) | 100 | (8) | | | | | | | 100 | (6) | 0 | (6) | *0.002* |
| AP Amplitude (mV) | 79.4 +/- 3.0 | (9) | 57.0 +/- 9.2 | (8) | *0.0002* | | | | | | 39.3 +/- 2.5 | (6) | NONE | (6) | |
| Threshold Potential (mV) | -58.7 +/- 1.7 | (9) | -53.9 +/- 2.2 | (8) | | | | | | | -49.5 +/- 3.4 | (6) | NONE | (6) | |
| Capacitance (pF) | 62.2 +/- 2.8 | (6) | 63.2 +/- 5.1 | (9) | | | | | | | | | | | |
| g-Leak (nS/pF) | 0.25 +/- 0.06 | (4) | 0.33 +/- 0.04 | (5) | | | | | | | | | | | |
| g-$Na_V$-Peak (nS/pF) | 0.78 +/- 0.08 | (9) | 0.85 +/- 0.10 | (9) | | 0.70 +/- 0.05 | (9) | 0.52 +/- 0.07 | (12) | | | | | | |
| Steady-State $Na_V$ Inactivation | | | | | | | | | | | | | | | |
| $V_{1/2}$ (mV) | -88.2 +/- 2.4 | (9) | -101.2 +/- 1.0 | (13) | *0.0001* | | | | | | | | | | |
| k (mV) | -4.3 +/- 0.3 | (9) | -4.7 + - 0.2 | (13) | | | | | | | | | | | |
| Tau I-$Na_V$ Decay at -35mV (ms) | 2.4 +/- 0.2 | (9) | 1.5 +/- 0.1 | (9) | *0.0002* | 1.6 +/- 0.1 | (9) | 1.0 +/- 0.1 | (13) | *0.006* | | | | | |
| g$Na_V$-Peak 9mV/ms Ramp (% g$Na_V$-Peak for Voltage Step) | 88.1 +/- 2.2 | (11) | 80.8 +/- 2.7 | (14) | *0.06* | 81.6 +/- 3.0 | (12) | 59.2 +/- 4.4 | (12) | *0.0003* | | | | | |

Supplementary Table 4: Recorded Cardiomyocyte Parameters. AP, action potential. Data represents mean ± s.e.m. Significant P values, Student's t-test, are shown for excitation and $Na_V$ parameter differences in *$Fhf2^{WT}$* versus *$Fhf2^{KO}$* cells.

| | 25°C | | | | | 35°C | | | | | 40°C | | | | |
|---|---|---|---|---|---|---|---|---|---|---|---|---|---|---|---|
| | $Na_V$1.5 + FHF2VY | | $Na_V$1.5 w/o FHF2 | | | $Na_V$1.5 + FHF2VY | | $Na_V$1.5 w/o FHF2 | | | $Na_V$1.5 + FHF2VY | | $Na_V$1.5 w/o FHF2 | | |
| **Parameter** | **mean +/- s.e.** | **# Cells** | **mean +/-s.e.** | **# Cells** | ***P (<)*** | **mean +/- s.e.** | **# Cells** | **mean +/-s.e.** | **# Cells** | ***P (<)*** | **mean +/- s.e.** | **# Cells** | **mean +/-s.e.** | **# Cells** | ***P (<)*** |
| Steady-State $Na_V$ Inactivation | | | | | | | | | | | | | | | |
| $V_{1/2}$ (mV) | -82.2 +/- 1.6 | (7) | -93.5 +/- 0.8 | (9) | *0.0002* | | | | | | | | | | |
| k (mV) | -8.6 + - 0.4 | (7) | -7.8 + - 0.4 | (9) | | | | | | | | | | | |
| Tau I-Nav Decay at -25mV (ms) | 0.63 +/- 0.03 | - (6) | 0.49 +/- 0.02 | (11) | *0.008* | 0.35 +/- 0.04 | (8) | 0.25 +/- 0.02 | (10) | *0.03* | 0.28 +/- 0.02 | (5) | 0.16 +/- 0.02 | (6) | *0.002* |
| g$Na_V$-Peak 9mV/ms Ramp (% g$Na_V$-Peak for Voltage Step) | 72.5 +/- 2.4 | (6) | 45.0 +/- 2.9 | (7) | *0.0001* | 40.0 +/- 2.8 | (6) | 14.0 +/- 2.7 | (6) | *0.0001* | 25.8 +/- 3.8 | (6) | 7.3 +/- 1.2 | (3) | *0.0006* |
| g$Na_V$-Peak 45mV/ms Ramp (% g$Na_V$-Peak for Voltage Step) | 99.4 +/- 1.5 | (6) | 92.3 +/- 2.2 | (7) | *0.02* | 90.8 +/- 2.2 | (6) | 70.9 +/- 5.4 | (6) | *0.01* | 82.6 +/- 4.6 | (6) | 48.5 +/- 7.1 | (5) | *0.005* |

Supplementary Table 5: Recorded HEK $Na_V$1.5 Parameters. Data represents mean ± s.e.m. Significant P values, Student's t-test, are shown for $Na_V$1.5 parameter differences recorded in HEK cells +/- FHF2VY expression.
